# Automatic Anomaly Detection on In-Production Manufacturing Machines Using Statistical Learning Methods

**DOI:** 10.3390/s20082344

**Published:** 2020-04-20

**Authors:** Federico Pittino, Michael Puggl, Thomas Moldaschl, Christina Hirschl

**Affiliations:** 1 Silicon Austria Labs GmbH, A-9524 Villach, Austria; Thomas.Moldaschl@silicon-austria.com (T.M.); Christina.Hirschl@silicon-austria.com (C.H.); 2 LAM Research AG, A-9524 Villach, Austria; michael.puggl@lamresearch.com

**Keywords:** anomaly detection, statistical machine learning, in-production data

## Abstract

Anomaly detection is becoming increasingly important to enhance reliability and resiliency in the Industry 4.0 framework. In this work, we investigate different methods for anomaly detection on in-production manufacturing machines taking into account their variability, both in operation and in wear conditions. We demonstrate how the nature of the available data, featuring any anomaly or not, is of importance for the algorithmic choice, discussing both statistical machine learning methods and control charts. We finally develop methods for automatic anomaly detection, which obtain a recall close to one on our data. Our developed methods are designed not to rely on a continuous recalibration and hand-tuning by the machine user, thereby allowing their deployment in an in-production environment robustly and efficiently.

## 1. Introduction

The onset of smart sensor networks and the Internet of Things (IoT) has opened up unprecedented possibilities in the industrial environment, leading to the surge of smart manufacturing and Industry 4.0. The ever-increasing amount of data enabled by such technologies paves the way for using Statistical Machine Learning (ML) methods to extract information from the data that would be impossible to obtain even by human experts. In this context, one of the most promising and relevant fields of interest is the derivation of models for anomaly and fault detection in manufacturing [1] and other industrial applications [2]. Automatic systems for anomaly detection can give relevant advantages to manufacturing companies by reducing their down-time due to machine breakdowns and by detecting a failure before this results in a catastrophic event, and this is enabled without the need for resorting to the work of expensive human experts in the field [1,2]. Popular methods for anomaly detection include both traditional control statistics, like the control chart [3,4,5,6,7,8], and machine learning (and most recently deep learning) methods [2,9,10,11,12], like for instance autoencoders [13], support vector machines [14], and convolutional and recurrent neural networks [15,16].

Despite the abundance of work in the literature on anomaly detection in manufacturing, we believe that there has been still insufficient investigation on the application of these techniques to in-production machines and in a realistic use-case. In particular, it is usually customary to monitor very long machine operations in a wide variety of operating conditions and with no anomalies, while examples of faults can usually be only artificially induced in a purpose-built demonstrator. This setting opens the question whether anomaly detection algorithms based on ML methods are actually able to predict faults in real conditions. In addition, the variability between different machines usually means that a delicate and expensive recalibration of the models might be often necessary, thereby increasing the overhead and the probability of model errors. Moreover, to the best of our knowledge, we think that a comparison between the control chart and anomaly classification methods based on ML in such a realistic use-case has not been properly addressed.

The paper is organized as follows: Section 2 presents in detail the machine under investigation and the collected data, going into detail on how the faulty data were obtained. Section 3 provides a detailed description of the algorithms that will be used for automatic anomaly detection. Section 4 then presents the results obtained with classical control charts, with a model based on the integration between control charts and ML classification algorithms and with a pure ML classification algorithm. Finally, Section 5 discusses the advantages and disadvantages of each model and draws our conclusions.

## 2. Context

**Figure 1:**
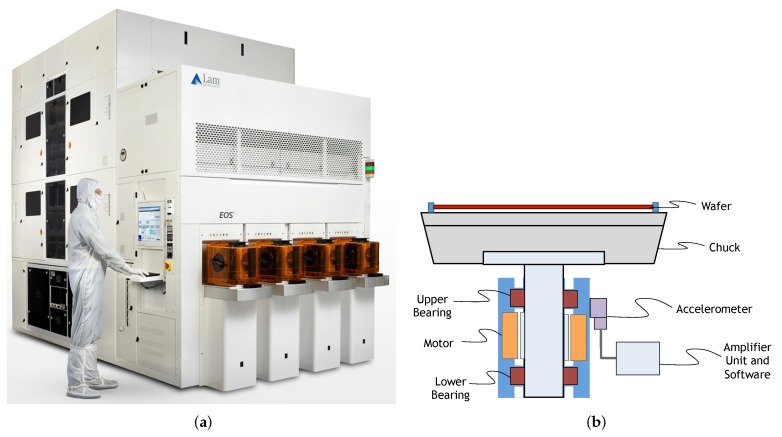
(**a**) Depiction of the full EOSmachine; (**b**) sketch of the Drive and Elevating Unit (DEU).

This work focuses on a machine for wet wafer processing in semiconductor manufacturing produced by LAM Research (Figure 1a), called EOS. The machine is made of either eight or 16 identical chambers, which process the incoming wafers in parallel. Within these chambers, the wafer is processed by either using reactive gasses or by treating its surface with liquid chemicals. Wet wafer cleaning is used between chip-processing steps to remove yield-limiting residues and defects. Continued device scaling, increasingly complex structures, and new materials bring additional defect removal challenges and also drive the need for increased wafer cleaning productivity.

Within this complex machine, this work focuses more specifically on the DEU (Drive and Elevating Unit), which is the main driver for the chuck (wafer clamping mechanism) in each chamber and is responsible for spinning the chuck and translating it in a vertical direction between each chamber level. It is a two-axis system, one axis providing rotational motion, the other axis providing vertical motion (Figure 1b). The two axes are driven by separate closed loop controlled servomotors and can therefore be operated independently. DEU condition monitoring aims at detecting any DEU failure early and therefore preventing wafers or machine damage, unscheduled tool down time, and/or yield impact. In the current version of this machine, the bearings on the DEU (see Figure 1b) are monitored via a vibration sensor; if the vibration level increases above some safety level, this indicates a failure on the bearing, and the machine must be stopped to prevent damage. Such a safety level is however only an upper bound for the fault, and it does not take into account the differences between chambers and the dependency of the average vibration level on the bearing’s rotation speed. The goal of this work is then to monitor the machine’s operation and to identify when the accumulated wear is high enough that the machine has to be stopped and undergo maintenance in order to prevent permanent damage.

The bearing’s vibration is measured (in module) through a piezoelectric accelerometer (Model PCB Piezotronics, M602D01 [17]) connected to an amplifier module (model Hansford Sensors HS535). The piezoelectric accelerometer signal is converted into the vibration acceleration peak-to-peak signal, which is then monitored online via EOS system software. Depending on which rotational speed the DEU is currently operating, specific manually-tuned limits for warning and error levels are defined. The data are then enriched with the rotation speed of the bearing at each time sample (measured in rpm) and finally centrally collected for further processing.

### Data Acquisition

**Figure 2:**
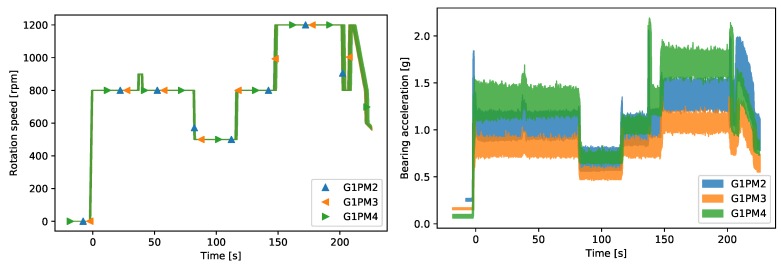
The figure shows all the time traces of the recorded speed (**left**) and vibrations (**right**) in each chamber (the different colors) of the LAB machine for all wafers. In the left plot, the lines overlap since all wafers run through the three chambers with the same speed profile.

For the purpose of anomaly detection, we first of all acquired a dataset that represented the normal operation of the DEU. We then chose one fully-functional EOS machine (in the following, we refer to this as the LAB machine), and we acquired data in three of its chambers, each one having a different level of mechanical wear. Approximately 85 wafers were processed in each chamber, and they were all operated with the same speed profile. Figure 2 shows all the time traces of the recorded speed and vibrations in each chamber (the different colors) for all wafers. The sampling rate was 5 Hz, and the total time spent by each wafer in the chamber was approximately 240 s. Depending on the particular process, sometimes, the wafers could also move vertically in the chamber, which was visible as the spike in acceleration at about 140 s in chamber G1PM4.

**Figure 3:**
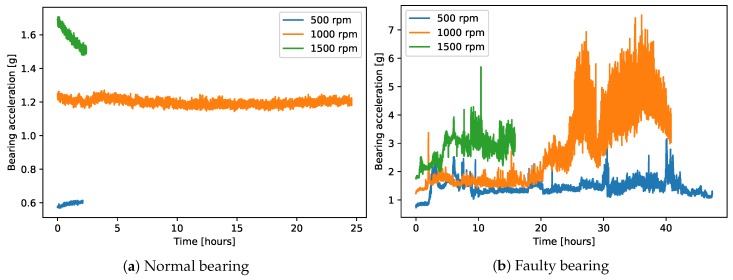
The figure shows the time traces of measured vibration for normal (**a**) and faulty (**b**) bearings in the demonstrator at different steady-state rotation speeds (the different colors). The normal bearing at 1000 rpm is measured for a longer time than the ones at 500 rpm and 1500 rpm to show the measurement stability in normal conditions over long time periods. Data were resampled to a 0.1 Hz sampling rate for better readability.

Given the nature of the mechanical system, faults on the bearing only occur after very long operations (up to several years); nevertheless, they can cause significant damage to the machine. Faults are normally caused by insufficient or absent lubricant in the machine over extensive time periods, which gradually damages the bearing. For these reasons, we decided to artificially induce faults on the machine by employing a normal and healthy bearing, but omitting the lubricant. In order to better characterize the faulty behavior, we built a demonstrator machine by mounting a chamber exactly equivalent to the ones of the LAB machine on a test bed. This setting allowed for better measurement control, for instance by directly measuring the bearing’s vertical displacement from the starting position, which would not be possible in the LAB machine. In the demonstrator, the bearings were continuously monitored in either normal or faulty conditions for much longer time frames, up to two days. The sampling rate was 2 Hz, the data is shown in Figure 3. In contrast with the measurements on the LAB machine, here the bearings were measured at steady state at various different rotation speeds, from 500 to 1500 rpm.

As the induced fault did not show immediate results on the measurement, any data collected before the evident failure of the bearing could be considered as normal. We did not however have any definite time instant when the fault happened, since we were observing a gradual wear process. From the measurement of bearing displacement, we could only identify the moment when the cumulative wear exceeded a certain security threshold, assumed at 0.4 mm displacement, or when the acceleration on the bearing exceeded a fixed safety threshold of 2*g* (the latter however only calibrated at 1000 rpm). Note that both of these hard thresholds were derived from mechanical considerations and experience from the machine’s manufacturer, but they did not represent a flexible and early way of detecting anomalies.

## 3. Methods for Anomaly Detection

Several different methods exist in the literature for anomaly detection. In order to classify all available methods, a very important distinction is the one between unsupervised and supervised methods [18], such a distinction having a great influence on the performance. The decision whether to choose one family of methods or the other is reliant on the nature of the available data, i.e., whenever a “ground truth” is not available, only unsupervised methods are possible. In the field of anomaly detection, it is often the case that only data describing normal operation are available. In such a case, only unsupervised methods are viable, because there is no example of an anomaly. Popular unsupervised methods include control charts based on clustering and one-class classification [7,19,20] or autoencoders [21,22,23]; see [8,12] for comprehensive reviews.

Moving towards supervised methods, yet still in the absence of anomalous data, control charts based on regression methods [8] are appropriate when the control variable is either derived from parameters that are not directly observable or from the difference between some estimated and measured variables. On the other hand, in the case that anomalous data are also available, fully supervised anomaly classification methods can be adopted, either based on classical ML algorithms, such as Support Vector Machines (SVM) or decision trees [8], or on deep learning, such as convolutional and recurrent neural networks [11,12].

In this paper, methodologies such as regression model control charts and anomaly classification will be adopted due to the peculiar nature of our data. The choice of the control chart was justified by the relative abundance of data for normal, with respect to anomalous, operation, which allowed for an accurate modeling of the control variable. The chosen regression algorithm was based on support vector machine [14], as this is a popular technique in anomaly detection [8], and was implemented using the LibSVM library [24] provided in the Python package scikit-learn. Other popular methods based on deep learning techniques, such as autoencoders [21,22,23], were not appropriate in our case since they require data to be both very abundant and highly multi-dimensional, which is a constraint that is usually not satisfied in industrial applications such as the one considered here.

As far as the anomaly classification is concerned, we again chose SVM both for similar reasons as before and to be able to compare the results with the control chart, while sharing the same underlying ML algorithm. The usage of an anomaly classification method can be more accurate than control charts as long as abundant anomaly data are available. Although this was not our case, we think that a comparison with the control chart was equally valuable in order to identify limits and differences between the two methods. Finally, although deep learning can be very effective also in anomaly classification [9,10,11,12,25], we believe it to be not applicable in our experiment, again due to the scarcity of data.

### 3.1. Control Chart

One of the most common approaches for statistical anomaly detection is based on the control chart [3], with applications ranging from industrial machines [5,8] to identifying contaminants [6] to farming [4] and multiple others. Contrary to most popular control chart approaches [8], in our case, we did not deal with multivariate data. The bearing’s vibration was, in fact, dependent on the rotation speed, and therefore, the two variables could not be assumed independent. The chosen approach was then to use a regression model control chart, deriving a regression model to estimate the expected vibration level given the set rotation speed. The control chart was then constructed on the regression residuals.

Several different methods have been presented in the literature for deriving regression model control charts [8], the most successful ones relying on ML algorithms. More recently, deep learning methods emerged as an evolution of the latter [9,11,12]; however, these usually require a greater amount of data for their derivation, being more suitable in the case of big data applications [10].

In order to derive the regression model, the input data were divided into windows, each 10s long, and in each window, five features were derived (average, standard deviation, maximum, minimum, kurtosis; see Table 1) for the rotation speed. The data were then divided into a training and a test set, using 80% of the wafers for training, as usual in machine learning procedures [18]. Finally, a Support Vector Regression (SVR) model [14] was used to regress the average of the vibration level in each window.

The second step for the creation of a control chart was to calculate the normalized relative errors (residuals) between the predicted and the measured average vibration in each window. In order to derive a statistical description of the normal population, the average and standard deviation σ of the residuals were computed on the training set. Finally, the control chart was displayed by showing the residuals together with their expected average, calculated on the training set. The anomaly was detected if the residuals exceeded such an expected average by more than a certain threshold, which was usually set at either 2σ or 3σ. In order to select such a threshold accurately, a 10-fold cross-validation was performed on the normal data, subdividing the original training set into validation and training sets.

### 3.2. Classification Methods

A completely different approach revolves around the usage of classification methods [18]. While a control chart can be derived without any anomaly example, classification needs to be performed in a purely supervised fashion, therefore requiring some sort of labeling of the data, which have to contain both normal and faulty operations.

Two types of classification strategies using support vector machines [14] are explored in this paper. The first strategy attempts to classify the type of chamber. As discussed in the previous section, the chambers were representative of three different levels of mechanical wear, and it was therefore important to identify the chamber correctly so that the appropriate predictive model could be used. Chamber classification was carried out with the same inputs as the control chart, but additionally calculating the windows’ features from Table 1 also on the measured vibration as additional inputs. For training, the same 80% of the wafers in each chamber used for the control chart were considered, and the rest left for testing. The data from the demonstrator were instead only used for testing, and this posed a challenge since we did not know which chamber was more representative of the mechanical wear state of the demonstrator.

As SVM was originally a binary classifier, a one-against-one approach [26] was adopted to accommodate multiclass classification. The second strategy attempted to classify the state of the bearing condition (i.e., normal or anomalous/faulty). Due to the use of an updated variant [27] of Platt’s scaling [28], the output of the SVM was a set of probabilities, which could be used to estimate the probability of failure. The outputs of the algorithm were then the probabilities $p_{it}$ of being in each of the three chambers (identified by index *i*). The predicted vibration at each window *t* was then calculated using a weighted average as:

(1) $$\begin{array}{c} {\tilde{v}}_{t}=\sum_{i}v_{it}p_{it} \end{array}$$

where $v_{it}$ is the predicted vibration for window *t* using the regression model from chamber *i*. The control charts’ average and standard deviations of the residuals were computed in the same way, for example:

(2) $$\begin{array}{c} \tilde{\sigma }=\sum_{i}p_{it}\sigma_{i} \end{array}$$

**Figure 4:**
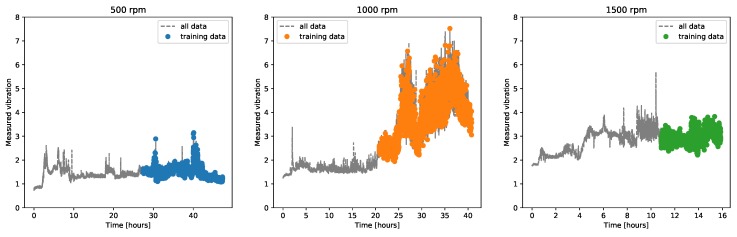
Anomaly data from the demonstrator, where also the part of the data used for training the SVM anomaly classifier is highlighted. The training data consisted also of part of the normal data from the LAB demonstrator (not shown in this Figure).

The second classification task aimed, instead, at directly estimating the probability of a failure, given a set input trace. The advantage of this approach with respect to the control chart was that it directly output an anomaly probability, and that, given its supervised nature, could be easily extend to multiple failure classes and severity. In our case, however, this was also a disadvantage, because our data consisted only of one failure measurement per rotation speed. For this reason, we decided to use as anomaly examples for the training phase the last part of the long time trace of the fault measurements (Figure 4). As normal examples, instead, the data from the LAB machine were used, coming from all three chambers. The input data were again transformed into the window features of Table 1, using exactly the same inputs employed in the chambers’ classification task, i.e., rotation speed and measured vibrations. The dataset was properly balanced in order to have a similar amount of faulty and normal time windows. Once again, the algorithm used was an SVM for consistency.

## 4. Results

### 4.1. Control Chart Using All Chambers’ Data

**Figure 5:**
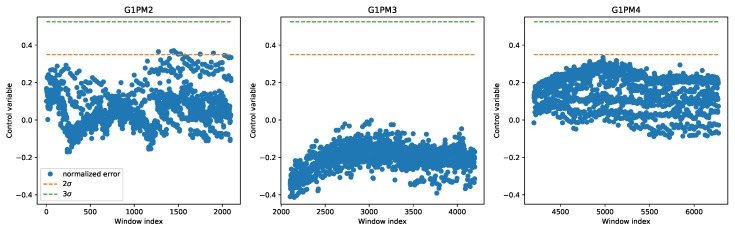
Control chart on the LAB machine derived with a single regression model for all chambers.

The first method we explored, as discussed in the previous section, was based on the control chart. In the first experiment utilizing the control chart, data from all three chambers were combined to train a single regression model. Figure 5 shows the control chart for all time windows in the LAB machine; the three chambers are clearly distinguishable by the different average vibration levels. In the control chart, only the upper limits are shown with the dashed line, both the 2σ and 3σ levels. Since in this case, all data were normal, they should all remain below the control chart limits. Using the 2σ level could lead to some outliers, but only in the first chamber. This finding showed very well the profound differences between the chambers.

**Figure 6:**
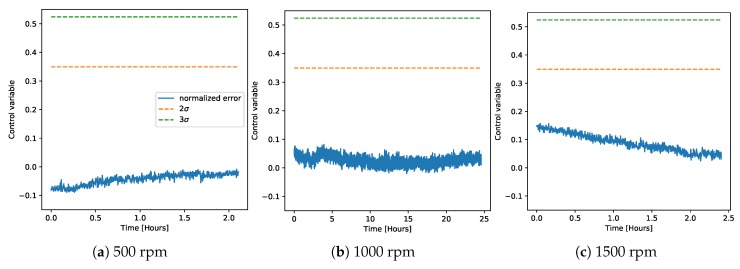
Control charts on the demonstrator derived using a single regression model trained on all chambers from the LAB machine. Here is shown the normal operation in the demonstrator at three different rotation speeds.

**Figure 7:**
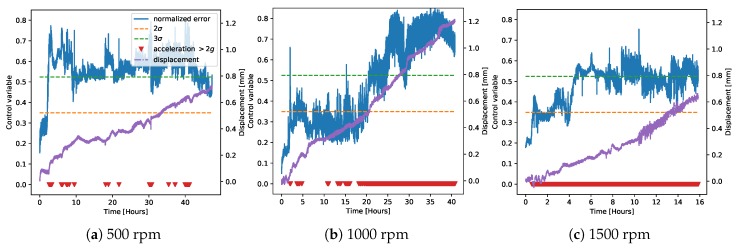
Control charts on the demonstrator derived using a single regression model trained on all chambers from the LAB machine, similar to Figure 6. Here is shown the faulty operation in the demonstrator at three different rotation speeds. The plots additionally show the points at which the measured value of acceleration is greater than 2g (red triangles), which corresponds to the hard threshold currently set in the machine to detect failure. The value of measured displacement is also shown (purple, scale on the right axis).

We then applied the trained regression model to derive a control chart on the data from the demonstrator. Figure 6 and Figure 7 show the control charts for both normal and faulty data on the demonstrator at three different rotation speeds. The plots additionally show the points at which the measured value of acceleration was greater than 2*g* (red triangles), which corresponded to the hard threshold currently set in the machine to detect failure, and the value of measured displacement for the faulty measurements (purple, scale on the right axis). The control chart was always able to classify the normal operation correctly. The faulty cases, instead, were usually easily distinguishable even at 2σ. The case at 1000 rpm was the one where the classification was more difficult, because the middle part of the time trace (between five and 20 h) did not look so clear as faulty operation. Since as explained in the previous section, the fault on the machine was induced by removing the lubricant, leading to a gradual wear rather than a point-wise fault, the behavior of the algorithm was consistent with these data. In fact, classifying as a fault only the beginning and the last part of the time trace showed that the bearing’s wear did not progress continuously, but rather had an initial spike, then a middle part where it almost stabilized, and a final part where the wear drastically increased again.

### 4.2. Control Chart With Per-Chamber Models

**Figure 8:**
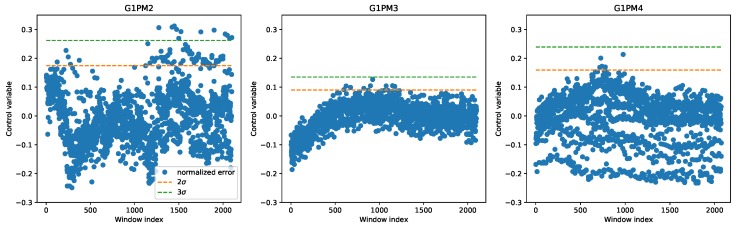
Control charts on the three chambers of the LAB machine derived using a different regression model in each chamber.

As we showed in the previous section, there existed relevant differences between the chambers. In order to obtain a more accurate representation of the wear state, it would be more appropriate to then derive three separate control chart models, one per each chamber. Such an approach allowed accounting for the variability between the chambers and to better detect an anomaly. Figure 8 shows the control charts on the LAB machine data (i.e., only normal operation), using for each chamber a regression model trained only on the data coming from the same chamber. In this case, as expected, we saw in all chambers some points above the 2σ level; however, we also observed some outliers at 3σ in chamber G1PM2. The possible explanation for these outliers was that the horizontal rapid movements that the wafers sometimes underwent in the production chambers could create peaks in the acceleration that were not related to any anomaly. On the other hand, on the demonstrator, since none of these horizontal movements occurred, no point fell above the threshold (see Figure 10).

**Figure 9:**
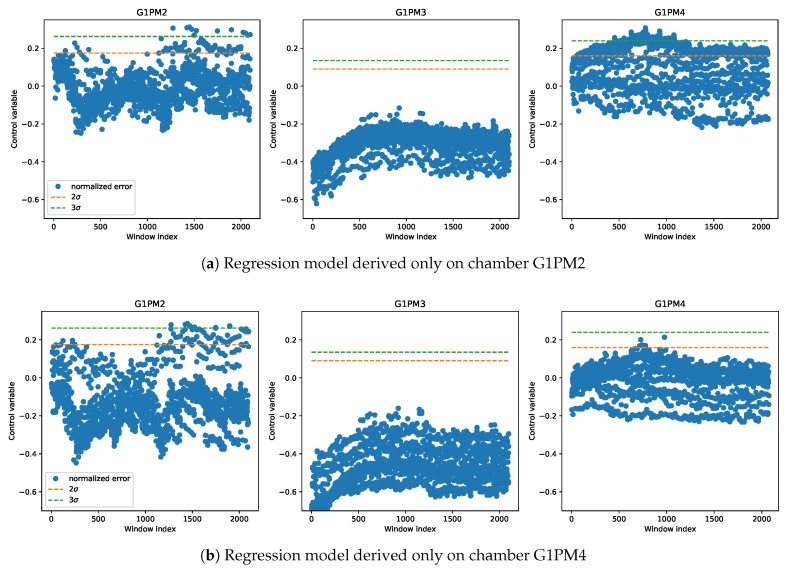
Control charts on the three chambers of the LAB machine using only one chamber to derive the regression model, which is then applied to derive the control chart in all chambers.

**Figure 10:**
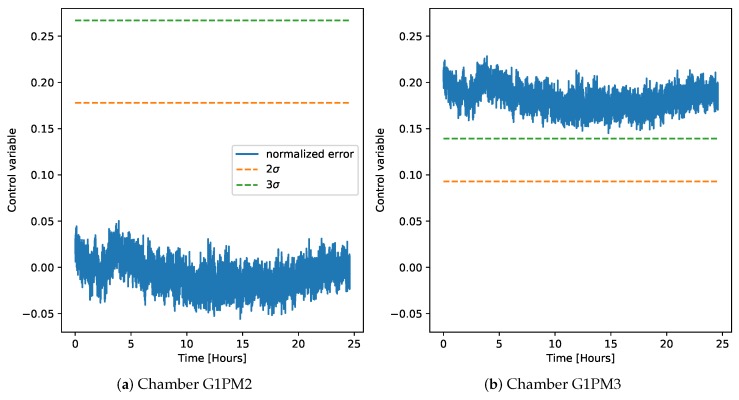
Control chart on the normal data from the demonstrator at 1000 rpm, using only one LAB machine chamber to derive the regression model. Here, the model based on chamber G1PM3 is shown instead of the one on chamber G1PM4 (as in Figure 9) to highlight that chamber G1PM3 is not suitable for the derivation of a reliable model.

Given that in a production scenario, it can be impractical to derive a regression model for each new chamber, there was a need to evaluate whether the model derived from on only one chamber could be reliably applied on the data coming from all other chambers. Figure 9 shows such a comparison when using either chamber G1PM2 (top) or G1PM4 (bottom) to derive the models. We note that the model’s performance could vary significantly based on the chamber selected for training. Moreover, it was clear that chamber G1PM3 had much less vibrations than the others due to the large margin between the model’s residuals and the thresholds in all cases, which could also be seen by comparing the average vibration levels in Figure 2. Since the three chambers represented different levels of mechanical wear, but still in the range of normal operation, a robust prediction model had to be able to classify data from all chambers correctly as normal. Using chamber G1PM3 for a model was therefore unlikely to satisfy this constraint and was for this reason unsuitable for deriving a robust model, as shown also in Figure 10. On the other hand, chamber G1PM4 was the one with the highest mechanical wear, and therefore, it showed the highest vibration level. For this reason, the model derived on this chamber was the only one that showed a good detection margin in all cases.

After the control chart models were derived on the LAB machine, we investigated their application on the demonstrator data. Figure 10 shows the derivation of the control chart on normal data, using as models either chamber G1PM2 (left) or G1PM3 (right). The model based on chamber G1PM3 is shown, instead of the one based on G1PM4, as in Figure 9, to verify the hypothesis that chamber G1PM3 was unsuitable for deriving a robust model, since its average vibration level was too small.

**Figure 11:**
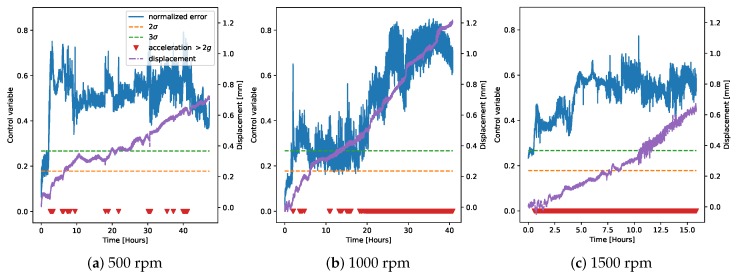
Control chart on the faulty data from the demonstrator, using only one LAB machine chamber (G1PM2) to derive the regression model, similar to Figure 10.

**Figure 12:**
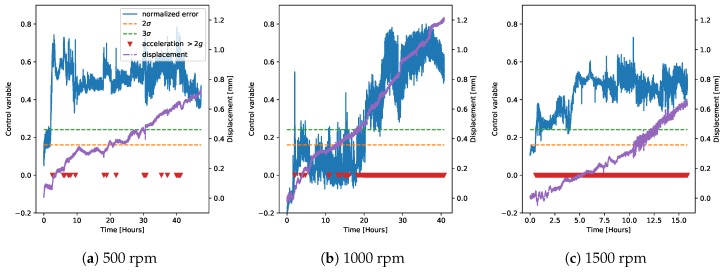
Control chart on the faulty data from the demonstrator, using only one LAB machine chamber to derive the regression model, as in Figure 10, but using chamber G1PM4 for the model.

We then decided to refrain from using the regression model derived on chamber G1PM3 and only to use the models derived on chambers G1PM2 and G1PM4 for deriving the control charts for faulty measurements on the demonstrator (Figure 11 and Figure 12). We note that at 500 rpm, the two models were very similar to each other and could easily identify the faulty operation. Moreover, the detection was very robust at 3σ, while the detection at 2σ delivered probably the best performance. At 1000 rpm, instead, the model on G1PM2 seemed the most appropriate, with the 2σ level being the most robust in this case. Finally, at 1500 rpm, the G1PM2 model might be too conservative. We should however note that the maximum rotation speed in the training data (on the LAB machine) was 1200 rpm, and therefore, inferring at 1500 rpm was not guaranteed to be extrapolated accurately.

### 4.3. Control Chart With Chamber Classification

**Figure 13:**
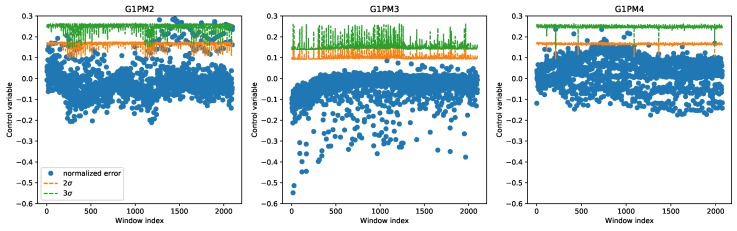
Control charts on the three chambers of the LAB machine derived using the weighted averages of the regression model predictions based on the chambers’ classification (Equation (2)).

The second method discussed in this work revolves around the derivation of a classification algorithm to select the appropriate control chart model to reflect the current state of the mechanical wear of our system, as discussed in Section 3. Figure 13 shows the application of this method to the normal data from the LAB machine. Here, the 2σ and 3σ levels were not constant because of the weighted average, as computed by Equation (2). The results looked, however, very consistent with the control charts derived on each of the chambers (Figure 9), therefore validating our approach for normal operation. The advantage here was that an automatic way of selecting the most appropriate model for each chamber was defined, and this was reflected in the correct margin between the average of the residuals and the control chart limits. In fact, focusing for instance on chamber G1PM3, in Figure 9, the distance between the vibration levels and the 2σ and 3σ thresholds was always very high, because of the much lower average vibration in this chamber, therefore not reflecting the operation of this particular chamber. In Figure 13, instead, also chamber G1PM3 is monitored with appropriate 2σ and 3σ levels.

**Figure 14:**
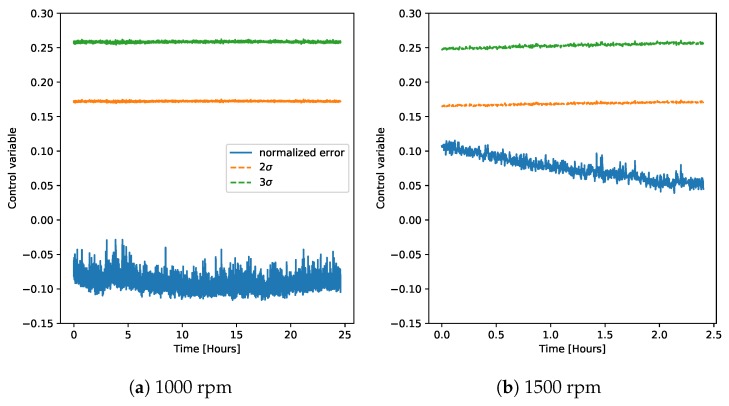
Control charts on the normal data from the demonstrator at different rotation speeds using the weighted averages of the regression model predictions based on the chambers; classification (Equation (2)).

Applying this method to the normal data on the demonstrator showed very similar results as the best cases before (Figure 14). The model weighted average performed very well in classifying the normal operation, despite the fact that the normal operation from the demonstrator data was not used for training, neither in the classification, nor in the control chart regression models.

**Figure 15:**
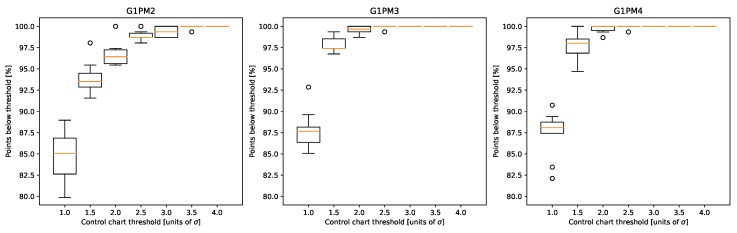
Investigation of the optimal threshold for the control charts using a 10-fold cross-validation. The data are derived from the three chambers of the LAB machine, and the control chart uses the weighted averages of the regression model predictions based on the chambers’ classification (Equation (2)).

As is evident from Figure 13, there was still however the need for selecting the most appropriate threshold to be used in the control chart. Fir this purpose, we performed a 10-fold cross-validation on the training data from the LAB machine. The training data were randomly divided into 10 distinct validation sets, and for each of these partitions, the control chart average and standard deviations were calculated on the rest of the training set. The validation set was then used to calculate the percentage of points where the residual error fell above a certain threshold, which was varied between 1 and 4 σ (Figure 15). Note that for both chambers G1PM3 and G1PM4, the 3 σ level never showed outliers, while for chamber G1PM2, as already noted, the threshold at 3 σ was sometimes not enough. Since however the number of outliers was on average only about 1% in this case, 3 σ could still be considered appropriate.

**Figure 16:**
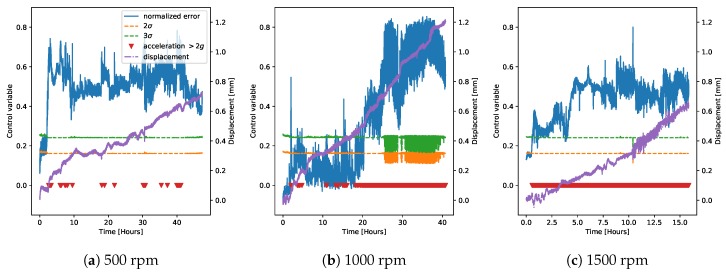
Control charts on the faulty data from the demonstrator at different rotation speeds using the weighted averages of the regression model predictions based on the chambers’ classification, similar to Figure 14.

Moving to the faulty data, Figure 16 compares the weighted average control charts to our baseline model (the one on chamber G1PM4; Figure 12). The performances of the two methods both at 500 rpm and at 1000 rpm were very similar. At 1500 rpm, instead, the model average tended to give more consistent results.

As a final consideration, note that the higher the displacement’s rate of change (the slope of the purple curves in Figure 16), the higher also the residual error, and therefore the higher the vibration level. This observation suggested that the distance of the residuals to the safety threshold could be used as an anomaly severity indication. In our case, however, the bearings were fully inspected only at the beginning and at the end of the measurement, while their status during the acquisition could only be inferred from the measured vibrations and displacement. For this reason, the derivation of a model for anomaly severity estimation was not possible with the currently available data and is left for future work.

### 4.4. Anomaly Classification

**Figure 17:**
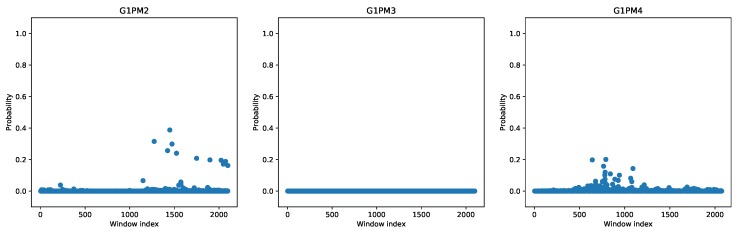
Anomaly classification results on the three chambers of the LAB machine.

The last anomaly detection algorithm we explored was based on a classification algorithm, as described in the previous section. For training, both the normal data on the LAB machine and the last few hours of the faulty data from the demonstrator were used. Figure 17 show the anomaly probability for the windows in the LAB machine. We observed that the classification accuracy was almost perfect, and very few points came close to the 0.5 anomaly probability.

**Figure 18:**
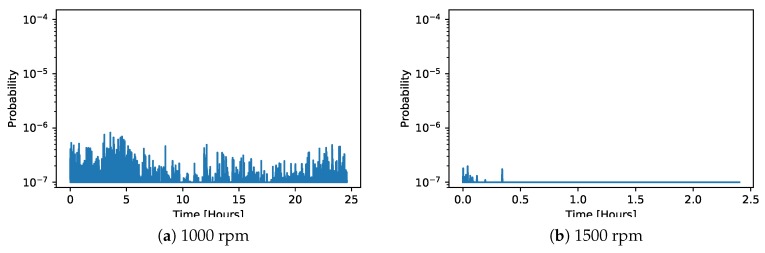
Anomaly classification results on the normal data from the demonstrator at different rotation speeds. Since the anomaly probability is very close to zero, the y-axis in this figure is logarithmic for better readability.

Figure 18 then shows the classification results on the normal data from the demonstrator, which was not used for training the classifier. In this case, the classification was perfect, with an anomaly probability extremely close to zero.

**Figure 19:**
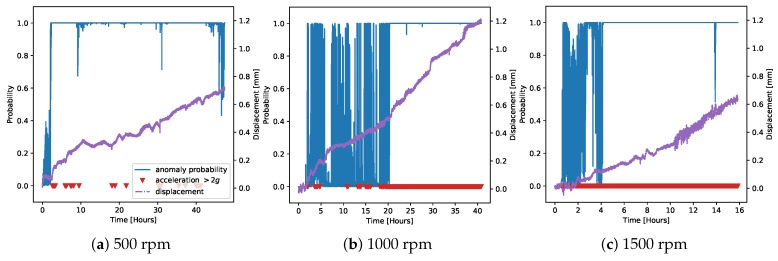
Anomaly classification results on the faulty data from the demonstrator at different rotation speeds.

Finally, Figure 19 shows the anomaly classification for the faulty data on the demonstrator, for all three measured rotation speeds. We immediately noted that the performance of this method was very similar to the one based on the control chart, despite them being two completely different models and using different training data. The uncertainty in the middle portion of the faulty data at 1000 rpm was observed in this case as well. These observations provided an important validation of our findings.

**Figure 20:**
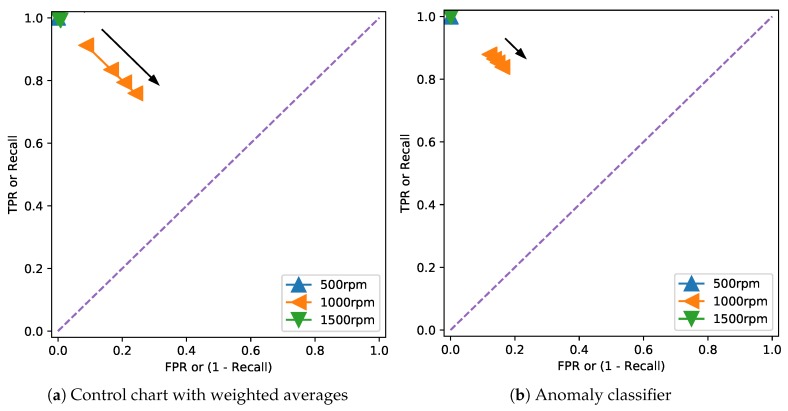
ROC curves of both the control chart and anomaly classification methods for the faulty data at the different rotation speeds. For the control chart, the changing parameter is the threshold on the residuals, ranging from 1 to 4 σ. In the anomaly classifier, the parameter is the probability threshold to flag the point as an anomaly, and it ranges from 0.2 to 0.8. In both cases, the arrows indicate the direction of increase in the threshold.

In order to provide a more precise comparison between the control chart and anomaly classification methods, Figure 20 shows the Receiver Operating Characteristic (ROC) curves [29] on the faulty data at the different rotation speeds. The recall was calculated by assuming that all points where the displacement was above 0.4 mm were anomalies, and the curve was obtained by varying the detection thresholds (on the residual error for the control chart, on the probability for the anomaly classifier). The performance at 500 and 1500 rpm in both cases was excellent, as already noticeable from Figure 16, Figure 17, Figure 18 and Figure 19. On the other hand, as noticed before, the measurement at 1000 rpm had a greater error due to the uncertainties in the middle part of the acquisition.

## 5. Conclusions

In conclusion, we performed a detailed investigation on different methods of automatically identifying anomalies in the operation of a rotating bearing in a commercial semiconductor manufacturing machine. We showed that both control charts and fully-supervised classification methods delivered a very similar performance, provided that the training data and the method were chosen appropriately. This finding was remarkable provided that the two methods used very different data, with the former not relying on anomaly examples, contrary to the latter. All these approaches proved able to provide a flexible and early way of detecting anomalies, with a much higher flexibility and robustness than setting a fixed threshold and reaching values of recall extremely close to one.

A successful definition of a method for anomaly detection was then highly dependent on the data available and not as much on the algorithmic choices. Approaches that did not rely on observing anomalies, like the ones based on the control chart, were more tailored to cases where fault examples were rare or absent, while fully-supervised approaches, like the ones based on anomaly classification, were more appropriate when the faults could be sufficiently characterized and distinguished.

We also showed that such methods could be reliably applied in an in-production industrial use-case, taking into account differences between machines with varying wear states. In fact, our automatic procedure could be calibrated once by the machines’ manufacturer by using machines with different wear states, without the need for continuous recalibration and hand-tuning by the machine user. The adaptation of the methods presented here to a real-time monitoring procedure was straightforward, giving an anomaly response every 10 s with the windows size chosen in this work. Such a response time was enough for this application since the anomalies resulted from a gradual process that needed long times to accumulate before it could cause a significant failure of the machine. We then believe that our results showed that the deployment of anomaly detection methods in manufacturing applications could be done robustly and efficiently, while providing relevant benefits.

## Figures and Tables

**Table 1:** Features used in the ML models, calculated on x(t), this being either vibration or rotation speed time traces, for each window *W* of length $N_{W}$.

Description	Definition
Average	$\bar{x}=E\left[x\left(t\right)\right]\frac{1}{N_{W}}\sum_{t\in W}x\left(t\right)$
Standard deviation	$\sigma_{x}=\sqrt{\frac{1}{N_{W}-1}\sum_{t\in W}{\left(x\left(t\right)-\bar{x}\right)}^{2}}$
Maximum	$x_{M}=\max_{t\in W}x\left(t\right)$
Minimum	$x_{m}=\min_{t\in W}x\left(t\right)$
Kurtosis	$x_{K}=E\left[{\left(\frac{x\left(t\right)-\bar{x}}{\sigma_{x}}\right)}^{4}\right]$
